# A Novel Interposer Channel Structure with Vertical Tabbed Vias to Reduce Far-End Crosstalk for Next-Generation High-Bandwidth Memory

**DOI:** 10.3390/mi13071070

**Published:** 2022-07-05

**Authors:** Hyunwoong Kim, Seonghi Lee, Kyunghwan Song, Yujun Shin, Dongyrul Park, Jongcheol Park, Jaeyong Cho, Seungyoung Ahn

**Affiliations:** 1 Korea Advanced Institute of Science and Technology, Cho Chun Shik Graduate School of Mobility, Deajeon 34051, Korea; khw3399@kaist.ac.kr (H.K.); ijhu12@kaist.ac.kr (S.L.); kyunghwan.song@kaist.ac.kr (K.S.); yujun.shin@kaist.ac.kr (Y.S.); dongryulpark@kaist.ac.kr (D.P.); 2 Department of System IC Development, National NanoFab Center, Daejeon 34141, Korea; jcpark@nnfc.re.kr; 3 Huwin, Seongnam 13558, Korea; jycho@huwin.co.kr

**Keywords:** broadside structure, far-end crosstalk, impedance, interposer channel, silicon interposer, vertical tabbed via

## Abstract

In this paper, we propose and analyze a novel interposer channel structure with vertical tabbed vias to achieve high-speed signaling and low-power consumption in high-bandwidth memory (HBM). An analytical model of the self- and mutual capacitance of the proposed interposer channel is suggested and verified based on a 3D electromagnetic (EM) simulation. We thoroughly analyzed the electrical characteristics of the novel interposer channel considering various design parameters, such as the height and pitch of the vertical tabbed via and the gap of the vertical channel. Based on the frequency-dependent lumped circuit resistance, inductance, and capacitance, we analyzed the channel characteristics of the proposed interposer channel. In terms of impedance, insertion loss, and far-end crosstalk, we analyzed how much the proposed interposer channel improved the signal integrity characteristics compared to a conventional structure consisting of micro-strip and strip lines together. Compared to the conventional worst case, which is the strip line, the eye-width, the eye-height, and eye-jitter of the proposed interposer channel were improved by 17.6%, 29%, and 9.56%, respectively, at 8 Gbps. The proposed interposer channel can reduce dynamic power consumption by about 28% compared with the conventional interposer channel by minimizing the self-capacitance of the off-chip channel.

## 1. Introduction

Recently, memory usage has steadily increased as technology has developed in the era of the fourth industrial revolution, with artificial intelligence, big data centers, robots, autonomous vehicles, augmented reality (AR), and virtual reality (VR). Representative memories are double data rate (DDR), graphics double data rate (GDDR), and high-bandwidth memory (HBM). In order to store and process these large amounts of data, demand for high-bandwidth memory has been rapidly increasing. HBM is the essential memory structure for industries in the fourth industrial revolution because it can implement high bandwidth more effectively than any other memory structure. HBM has a 3D stacked structure based on through-silicon vias (TSVs). The development of silicon interposer interconnection technologies, such as interposer channel and TSV, are essential for the development of next-generation HBM and chip-to-chip interfaces [1,2]. HBM is designed as a parallel interface structure with 1024 IOs [3].

To meet the needs of the next generation of HBMs, it is essential to address the issue of signal integrity. Signal integrity needs to be improved to resolve issues such as channel loss, crosstalk, impedance mismatching, and inter-symbol interference (ISI).

The HBM interconnection consists of the TSV, interposer channel, pad, package, etc. Because the interposer channel has the longest physical channel length in the off-chip interface, it must be considered and analyzed in terms of signaling within the target frequency band. Various channel components such as the interposer channel effectively have low-pass filter characteristics, so signal degradation occurs as the frequency increases.

**Figure 1:**
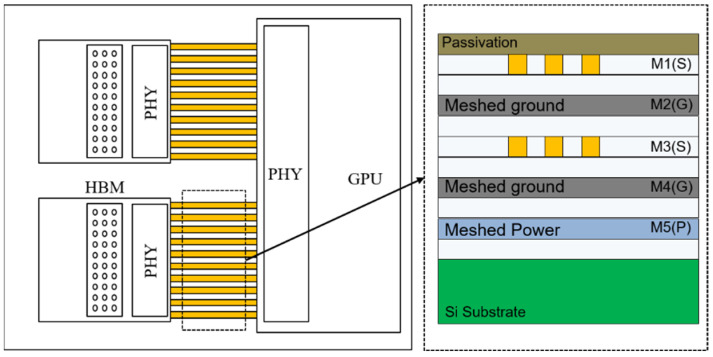
Conventional HBM interposer channel.

The conventional HBM interposer channel has three problems, as shown in Figure 1. Firstly, the HBM interposer channel has a lower impedance than the transmitter (TX) and receiver (RX) driver. In general, although the dielectric can be manufactured as thin as about 1 μm, it is possible to manufacture a relatively wide channel with a width of at least 2 μm or more due to the limitations of the semiconductor manufacturing process. Since the channel is relatively wide, the impedance formed is inevitably very small. In [4], in order to increase the impedance of the interposer channel, the ground slot under the signal channel was presented. Secondly, from the paper [5], the power consumption of an off-chip interconnection was found to be generally 100 times larger than that on-chip. Thus, when designing an off-chip interconnection, it is essential to consider the low power consumption. The third HBM interface problem is the very high channel density, due to the 1024 IO channels. If the clock and address line are combined, a very large number of channels need to be placed. There is not enough free space. Therefore, a solution to the HBM interposer channel structure is required. The new interposer channel solution should alleviate the influence of impedance discontinuity and minimize the issue of off-chip power consumption, while not causing a routing issue.

Regarding signal integrity, previous research on the HBM interface has been carried out in various ways, which can be divided into several categories [4,6,7,8,9,10]. Refs. [4,6] present the signal integrity design method and the analyses of conventional interposer channels such as the micro-strip and strip line. There has been an insufficient number of suggestions for methods to improve the signal integrity characteristics of the interposer channel itself. Refs. [7,8] are related studies, proposing and analyzing passive equalizers. The passive equalizer can minimize ISI and flatten channel loss by intentionally leaking a low-frequency band signal. However, it requires additional chip area. Because the HBM interposer channel is already very dense, it is not realistic for application to the HBM. Refs. [9,10] report the on-chip solutions. The representative on-chip solutions are the crosstalk reduction circuit method and data bus inversion (DBI) coding. These methods can effectively minimize the influence of far-end crosstalk noise. However, these methods require an additional circuit and can cause additional power consumption. Moreover, it is not possible to fundamentally improve the cause of channel loss in the case of impedance mismatching, reflection, etc. With DBI coding, a DBI channel is required, so issues with space also occur. Ref. [11] studies the structure of inserting the shielding channel in the interposer. Shielding channels can reduce crosstalk between channels. However, it can generate unnecessary self-capacitance, causing the impedance mismatching issue. Furthermore, inserting shielding channels incurs additional costs and requires more space. Thus, it is not a suitable method for the HBM interface.

In this paper, a novel broadside interposer channel structure with a vertical tabbed via for high-speed signaling and low-power consumption is proposed for next-generation HBM. The broadside structure solves the problem of low impedance and the issue of off-chip interconnection power consumption. The proposed structure uses the vertical tabbed via to mitigate the far-end crosstalk of the broadside structure. The proposed channel structure is presented and analyzed based on the modeling of self-capacitance and mutual capacitance. We also take into account the effect of design parameters such as the physical dimensions of the vertical tabbed via and evaluate it from a signal integrity perspective. The signaling performance of the proposed channel structure was analyzed for impedance, channel loss, far-end crosstalk, and eye-diagram compared to a conventional channel structure that includes the micro-strip line and strip line. We also evaluated the dynamic power consumption and compared it with a that of a conventional channel structure.

## 2. Proposal and Modeling of the Broadside Interposer Channel Structure with a Vertical Tabbed Via for Next Generation HBM

### 2.1. The Structure of the Proposed Novel Broadside Interposer Channel Structure

**Figure 2:**
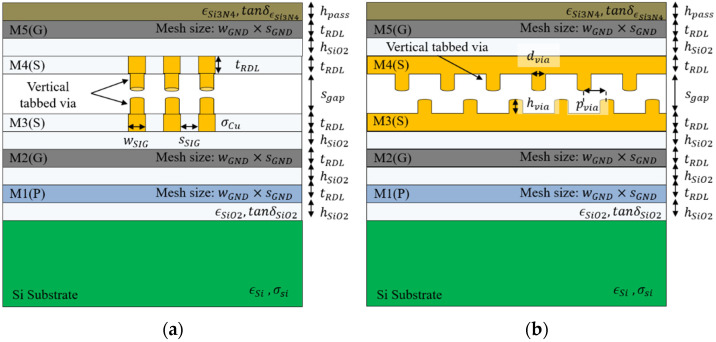
The proposed interposer channel structure: (**a**) front view and (**b**) side view.

The proposed channel structure is shown in Figure 2. The proposed channel structure has two features. First, the proposed channel structure is a broadside strip line channel structure. This broadside structure can minimize channel loss by reducing impedance mismatching. In addition, if a multi-channel system is required that is more than three signal layers, the proposed broadside channel structure can be designed with a high channel density structure because it can minimize the exposure of E-field and H-field to the outside. This will be detailed in Section 4.

Second, the proposed channel structure has a vertical tabbed via. The vertical tabbed via can mitigate the influence of far-end crosstalk between vertical channels. This effect is similar to the effect of reducing far-end crosstalk by increasing mutual capacitance, such as tabbed routing mainly used in the breakout zone of PCBs [12,13]. The physical dimension and electrical properties are summarized in Table 1. The interposer channel width and space are assumed to be 3 μm and 3 μm, respectively, and the silicon conductivity is 10 σ/m.

### 2.2. Analytical Modeling of Self and Mutual Capacitance for the Proposed Interposer Channel Structure

The electrical characteristics of the proposed channel structure were analyzed depending on the design parameters by building an analytical model of self-capacitance and mutual capacitance.

**Figure 3:**
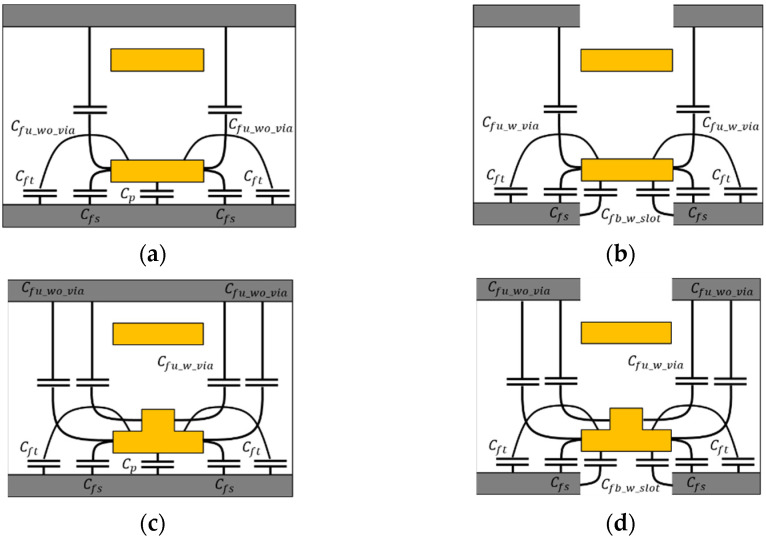
Cross-section view of self-capacitance of the proposed channel structure. (**a**) section 1: without the vertical tabbed via and without the ground slot, (**b**) section 2: without the vertical tabbed via and with the ground slot, (**c**) section 3: with the vertical tabbed via and without the ground slot, (**d**) section 4: with the vertical tabbed via and with the ground slot.

The self-capacitance of the proposed channel structure can be calculated by dividing it into four sections based on the cross-section, as shown in Figure 3. The four sections were determined according to the ground slots and the vertical tabbed via. The self-capacitance consists of various fringing capacitances between the bottom ground layer and the top ground layer. The equations of fringing capacitances for self-capacitance are expressed through (1)–(17) [14,15,16,17,18].

The $C_{p}$ is simply determined using parallel-plate capacitor formulas. The $C_{fs}$ is the analytical expression of external fringing capacitance. The effective thickness of the substrate is determined depending on the height of the $s_{gap}$. The $C_{ft}$ is calculated by (3) valid at the range 0.5 ≤ $h_{SiO2}$/$t_{RDL}\;$≤ 4 [15]. From the equations in [15], the factor of $A_{0}$, $B_{0}$,$\;C_{0}$ for $C_{ft}$ can be calculated. The $C_{fu\_wo\_via}$ and $C_{fu\_w\_via}$ are also the external fringing capacitances between channel and ground capacitance [16]. The $C_{fu\_w\_slot}$ is the internal fringing capacitance, which is unlike external fringing capacitance, since the electric field can be distorted into a non-perfect parabola shape [16].

For section 2 and section 4 with a ground slot due to the meshed ground layer, the total capacitance of those sections was calculated, including the $C_{fb\_w\_slot}$ generated from the four sides, as shown in Figure 3b,d. For section 3 and section 4 with the vertical tabbed via, the total capacitance of those sections was calculated, including the $C_{fu\_w\_via}$ from two sides, as shown in Figure 3c,d.

(1) $$C_{p}=\varepsilon_{SiO{}_{2}}\varepsilon_{0}\frac{w_{SIG}}{h_{SiO2}}$$

(2) $$C_{fs}=\frac{\varepsilon_{SiO{}_{2}}\varepsilon_{0}}{\pi }\left[2t_{b1}\ln\left(t_{b1}+1\right)-\left(t_{b1}-1\right)\ln\left(t_{b1}^{2}-1\right)\right]$$

(3) $$C_{ft}=\frac{\varepsilon_{SiO{}_{2}}\varepsilon_{0}e^{\left(-\frac{A_{0}}{B_{0}}\right)}\left[\ln\left(1+\frac{2w_{SIG}}{S_{SIG}}\right)+e^{\left(-\frac{C_{0}}{-3S_{SIG}}\right)}\right]}{\varepsilon_{SiO{}_{2}}\varepsilon_{0}e^{\left(-\frac{A_{0}}{B_{0}}\right)}+A\left[\ln\left(1+\frac{2w_{SIG}}{S_{SIG}}\right)+e^{\left(-\frac{C_{0}}{-3S_{SIG}}\right)}\right]}$$

(4) $$C_{fu\_wo\_via}=\frac{\varepsilon_{SiO{}_{2}}\varepsilon_{0}}{\pi }\left[2t_{b\_wo\_via}\ln\left(t_{b\_wo\_via}+1\right)-\left(t_{b\_wo\_via}-1\right)\ln\left(t_{b\_wo\_via}^{2}-1\right)\right]$$

(5) $$C_{fb\_w\_slot}=\frac{\varepsilon_{SiO{}_{2}}\varepsilon_{0}}{\pi }A_{1}\left[\frac{s_{SIG}}{2h_{SiO{}_{2}}+t_{RDL}}-\frac{2}{\pi }\ln\left(\frac{\pi }{2}\frac{s_{SIG}}{h_{SiO{}_{2}}+t_{RDL}}\right)\right]+B_{1}$$

(6) $$C_{fu\_w\_via}=\frac{\varepsilon_{SiO{}_{2}}\varepsilon_{0}}{\pi }\left[2t_{b\_w\_via}\ln\left(t_{b\_w\_via}+1\right)-\left(t_{b\_w\_via}-1\right)\ln\left(t_{b\_w\_via}^{2}-1\right)\right]$$

(7) $$C_{section1}=C_{p}+2C_{fs}+2C_{ft}+2C_{fu\_wo\_via}$$

(8) $$C_{section2}=4C_{fb\_w\_slot}+2C_{fs}+2C_{ft}+2C_{fu\_wo\_via}$$

(9) $$C_{section3}=C_{p}+2C_{fs}+2C_{ft}+2C_{fu\_w\_via}+2C_{fu\_wo\_via}$$

(10) $$C_{section4}=4C_{fb\_w\_slot}+2C_{fs}+2C_{ft}+2C_{fu\_w\_via}+2C_{fu\_wo\_via}$$

(11) $$C_{self}=C_{section1}+C_{section2}+C_{section3}+C_{section4}$$

where

(12) $$t_{SIG\_eff}=\frac{t_{RDL}+s_{gap}}{6}$$

(13) $$t_{b1}=\frac{2h_{SiO{}_{2}}+t_{SIG\_eff}}{2h_{SiO{}_{2}}}$$

(14) $$h_{wo\_via}=s_{gap}+2h_{SiO{}_{2}}$$

(15) $$h_{w\_via}=s_{gap}+2h_{SiO{}_{2}}-h_{via}$$

(16) $$t_{b\_wo\_via}=\frac{2h_{wo\_via}+t_{SIG}}{2h_{wo\_via}}$$

(17) $$t_{b\_w\_via}=\frac{2h_{w\_via}+t_{RDL}}{2h_{w\_via}}$$

(18) $$C_{s\_wo\_via}=\varepsilon_{SiO{}_{2}}\varepsilon_{0}\frac{w_{SIG}}{s_{gap}}$$

(19) $$C_{s\_w\_via}=\varepsilon_{SiO{}_{2}}\varepsilon_{0}\frac{w_{SIG}}{s_{gap}-h_{via}}$$

(20) $$C_{f\_wo\_via}=0.5\frac{\varepsilon_{r}}{60c_{0}\pi }\left[\frac{K\left(k_{oi1}\right)}{K\left(k_{oi1}^{\prime }\right)}-\frac{K\left(k_{ei1}\right)}{K\left(k_{ei1}^{\prime }\right)}\right]$$

(21) $$C_{f\_w\_via}=0.5\frac{\varepsilon_{r}}{60c_{0}\pi }\left[\frac{K\left(k_{oi2}\right)}{K\left(k_{oi2}^{\prime }\right)}-\frac{K\left(k_{ei2}\right)}{K\left(k_{ei2}^{\prime }\right)}\right]$$

(22) $$C_{section\_A}=C_{s\_wo\_via}+2C_{f\_wo\_via}$$

(23) $$C_{section\_B}=C_{s\_w\_via}+2C_{f\_w\_via}$$

(24) $$C_{mutual}=C_{section\_A}+C_{section\_B}$$

where

(25) $$k_{oi1}=\tanh\left(\frac{\pi }{4}\frac{w_{SIG}}{s_{SIG}}\right)/tanh\left(\frac{\pi }{4}\frac{s_{gap}+w_{SIG}}{s_{SIG}}\right)$$

(26) $$k_{oi2}=\tanh\left(\frac{\pi }{4}\frac{w_{SIG}}{s_{SIG}}\right)/tanh\left(\frac{\pi }{4}\frac{s_{gap}-h_{via}+w_{SIG}}{s_{SIG}}\right)$$

(27) $$k_{ei1}=\tanh\left(\frac{\pi }{4}\frac{w_{SIG}}{s_{SIG}}\right)tanh\left(\frac{\pi }{4}\frac{s_{gap}+w_{SIG}}{s_{SIG}}\right)$$

(28) $$k_{ei2}=\tanh\left(\frac{\pi }{4}\frac{w_{SIG}}{s_{SIG}}\right)tanh\left(\frac{\pi }{4}\frac{s_{gap}-h_{via}+w_{SIG}}{s_{SIG}}\right)$$

(29) $$k\prime_{oi1}^{2}=1-k_{oi}^{2}$$

(30) $$k\prime_{oi2}^{2}=1-k_{o2}^{2}$$

(31) $$k\prime_{ei1}^{2}=1-k_{ei}^{2}$$

(32) $$k\prime_{ei2}^{2}=1-k_{e2}^{2}$$

**Figure 4:**
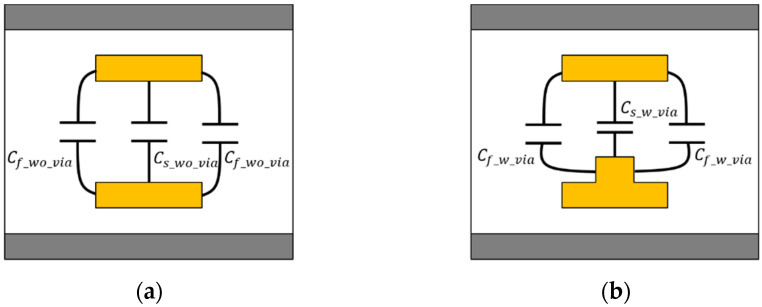
Modeling of mutual capacitance of the proposed channel structure. (**a**) Section A: without the vertical tabbed via, (**b**) Section B: with the vertical tabbed via.

The mutual capacitance of the proposed structure can be calculated by dividing it into two sections based on the cross-section, as shown in Figure 4. In mutual capacitance, unlike in self-capacitance, the effect of the ground slot is negligible. The equation of fringing capacitance for mutual capacitance is expressed through (18)–(32) [17]. The $C_{s\_wo\_via}$ and $C_{s\_w\_via}$ are simply determined by the parallel-plate capacitor formula. The $C_{f\_wo\_via}$ and $C_{f\_w\_via}$ are an empirical gap capacitance in the dielectric [19]. The *K*(*k*) and *K*(*k*′) are the complete elliptic integral of the first type and its complement.

**Figure 5:**
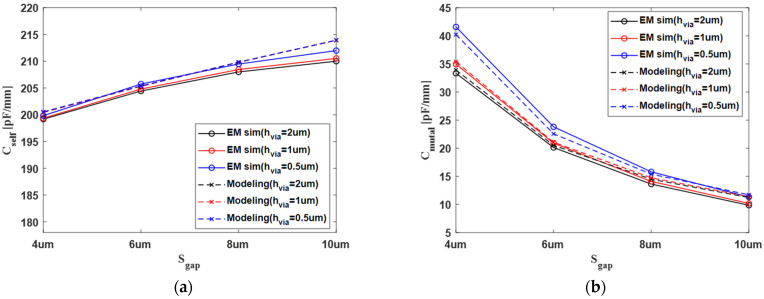
Verification of capacitance modeling: (**a**) self-capacitance and (**b**) mutual capacitance.

For the design parameters for $s_{gap}$ and $h_{via}$, the analytical modeling of the self-capacitance and mutual capacitance was verified based on 3D electromagnetic (EM) simulation, using the ANSYS Q3D 3D field simulator, as shown in Figure 5. With the variation in $s_{gap}$ and $h_{via}$, the trend in the capacitance model for the analytical modeling and 3D EM simulation is overall very similar, and the error is within 5%. As the $s_{gap}$ increases, the electric field is more strongly coupled to the ground layer of M2, which is close to the target channel of M3. However, the electric field between the M5 ground layer is relatively weak. Therefore, the total self-capacitance value eventually increases because the increase in the fringing capacitance between the ground layer of M2 close to the target channel of M3 is larger than the decrease of the fringing capacitance between the ground layer of M5 farther away from the target channel of M3. As the height of the vertical tabbed via increases with the same vertical distance of channel, it can be seen that the mutual capacitance significantly increases.

When the $s_{gap}$ has a value of about 10 μm, the self-capacitance has a saturated constant value. The 10 μm is a sufficiently wide gap of the channel because it is 10 times the thickness of the channel. Thus, this modeling of capacitance is presented with sufficient height.

## 3. Signal Integrity Analysis of the Proposed Interposer Channel Structure Depending on the Design Parameters

In terms of signal integrity, the characteristics of the proposed channel structure were analyzed depending on the design parameters that can affect the channel performance of the proposed structure. The influence of far-end crosstalk was evaluated through the power-sum far-end crosstalk (PSFEXT) [20]. The PSFEXT can consider all the effects of surrounding channels that include top and bottom aggressor channels for one victim channel. The PSFEXT equation is expressed in (33). The channel length is set to 5 mm. This channel length assumes the longest interposer channel length of the HBM interface.

**Figure 6:**
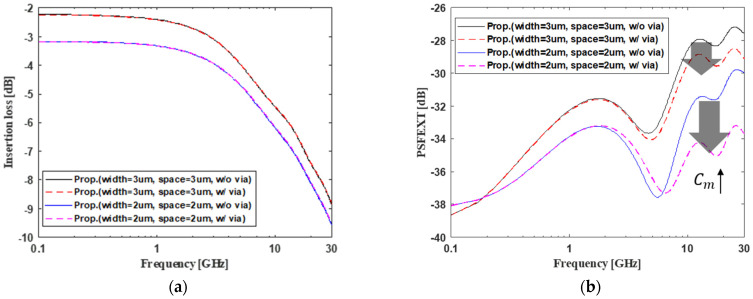
Signal integrity analysis of the effect of the proposed channel structure on the vertical tabbed via in the frequency domain. (**a**) Insertion loss and (**b**) power sum far-end crosstalk (PSFEXT).

First, the proposed channel structure was analyzed depending on the presence of a vertical tabbed via. Based on the analytical capacitance modeling, the self-capacitance of the proposed channel with the vertical tabbed via was not dramatically different from the proposed channel structure without the vertical tabbed via. Therefore, there was no significant change in terms of channel loss, as shown in Figure 6a.

On the other hand, when the structure had the vertical tabbed via, the amount of mutual capacitance could increase significantly, based on the analytical modeling. The FEXT coefficient can be expressed in (34). When the input signal transitions from low to high, the FEXT is a negative signal that has a 180-degree phase with the input signal. The FEXT coefficient is negative using (34). Therefore, in order to reduce FEXT, it is necessary to increase the mutual capacitance or reduce the self-capacitance.

The mutual capacitance is increased by inserting the vertical tabbed via, so the PSFEXT can be greatly mitigated, as shown in Figure 6b. In addition, looking at the PSFEXT, capacitive coupling is dominant above 1 GHz, and inductive coupling is dominant below 1 GHz [6]. The increase in mutual capacitance produced by inserting the vertical tabbed via, confirms that the FEXT is greatly reduced only above 1 GHz. In the case of width and space of 3 μm and 2 μm, respectively, there is a frequency band with large FEXT when the vertical tabbed via is inserted. This is because the resonance frequency occurs due to the cable length. If the cable is long, the channel should be designed considering the cable resonance.

(33) $$Powersum\;FEXT\;\left(PSFEXT\right)=10\log\underset{j\in \Omega_{FEXT}}{\sum }{\left|S_{i,j}\right|}^{2}$$

(34) $$FEXT=\frac{len_{coupling}}{2vT_{r}}\left(\frac{C_{m}}{C_{s}}-\frac{L_{m}}{L_{s}}\right)$$

Three design parameters of the proposed channel structure were analyzed, the height and the pitch of the vertical tabbed via and the gap of the vertical channel. Next, we analyzed the far-end crosstalk. As mentioned before, the insertion loss did not significantly change depending on the presence of the vertical tabbed via.

**Figure 7:**
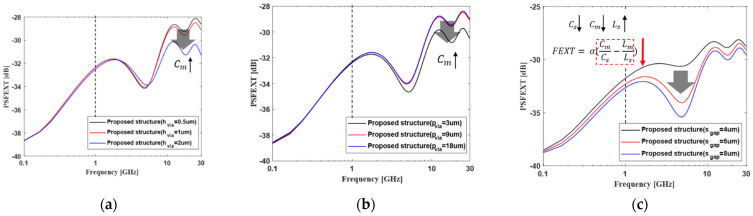
The PSFEXT analysis of proposed channel structure depending on the design parameters. (**a**) The height of the vertical tabbed via, (**b**) the pitch of the vertical tabbed via, and (**c**) the gap of the vertical channel.

As shown in Figure 7a, as the height of the vertical tabbed via increases, the mutual capacitance that occurs between the channels increases. Thus, the far-end crosstalk can be reduced. If the pitch of the vertical tabbed via becomes narrow, the far-end crosstalk can be reduced in the same way, as shown in Figure 7b. As the gap of the vertical channel increases, the far-end crosstalk decreases because the physical channel distance increases, as shown in Figure 7c. In this case, since mutual inductance also decreases, the far-end crosstalk is reduced even below 1 GHz. In the above, at 1 GHz, the value of the FEXT coefficient decreases because the mutual capacitance decreases while the self-capacitance and self-inductance increase. It is important to note that, as the gap in the vertical channel increases, the difference in far-end crosstalk improvement also decreases. That is, when the gap of the vertical channel becomes larger than a certain value, the far-end crosstalk can be saturated.

## 4. Signal Integrity Analysis of the Proposed Interposer Channel Structure Compared with the Conventional Interposer Channel Structure for the HBM

### 4.1. Signal Integrity Analysis of the Proposed Novel Interposer Channel Structure

**Figure 8:**
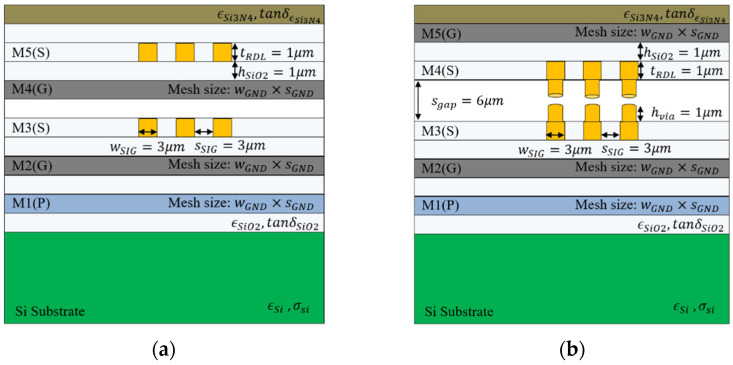
Interposer structure: (**a**) conventional channel structure and (**b**) proposed channel structure.

The signaling performance of the proposed channel structure and conventional channel structures, that is the micro-strip line and strip line, can be compared in terms of signal integrity. The interposer channel was selected as a five-layer structure, including two signal layers, two ground layers, and one power layer, as shown in Figure 8 [6]. The design parameters of the vertical tabbed via were selected to be 6 μm for $s_{gap}$, 1 μm for $h_{via}$, and 9 μm for $p_{via}$ based on the analysis results in the previous chapter. Both the conventional channel structure and the proposed channel structure were placed in a perfectly misaligned arrangement with a meshed ground layer. Since the impedance mismatching is minimized at the position where the signal channel and the meshed ground are perfectly misaligned, the channel loss is less degraded, and the eye margin can be greatly secured [6].

**Figure 9:**
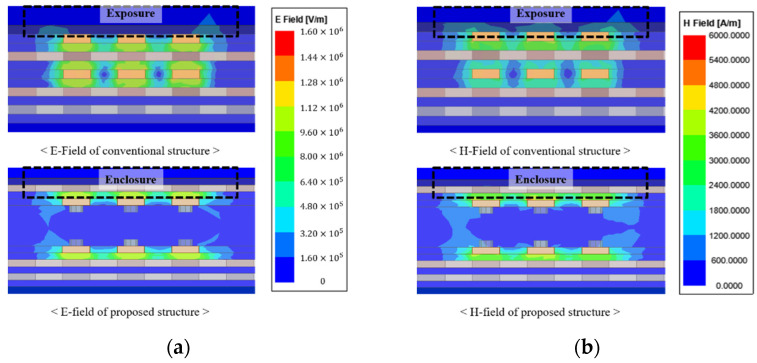
Field distribution of the proposed channel structure and conventional channel structure: (**a**) E-field and (**b**) H-field.

The E-field and H-field distribution are shown in Figure 9. It can be seen that the E-field and H-field are widely exposed because the micro-strip line does not have a ground layer above the channel. On the other hand, the proposed channel structure can minimize field distribution to the outside by arranging the ground layer above and below. In this case, if an additional signal layer is placed, one signal layer and one ground layer are required in the conventional channel structure. However, since the proposed channel structure has a ground layer on the top and bottom to minimize exposure to the outside, only the one signal layer is required. After all, in a system that requires many signal layers, if one layer can be reduced, an advantage in channel density can be obtained.

**Figure 10:**
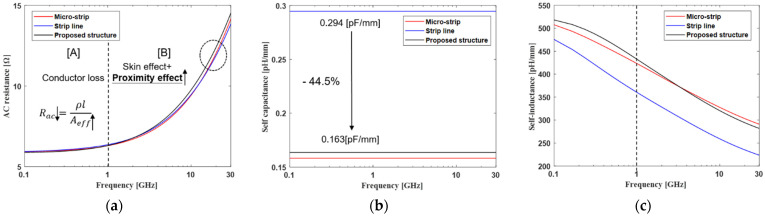
RLC component of the proposed channel structure: (**a**) AC resistance, (**b**) self-capacitance, and (**c**) self-inductance.

Figure 10 shows an RLC component of the proposed channel structure and conventional channel structure based on the 3D EM simulation, using the ANSYS HFFF 3D field simulator. Since the cross-sectional area of the conductor through which current flows is as large as the area of the vertical tabbed via compared to the conventional channel structure, the conductor loss of the proposed channel structure is smaller than that of the conventional channel structure below 1 GHz, as shown in Figure 10a. In general, above 1 GHz, the AC resistance is determined by the skin effect and proximity effect. The proposed channel structure has a larger AC resistance compared to the conventional channel structure because the proximity effect increases as the distance between the adjacent channels decreases, due to the vertical tabbed via. Additionally, due to chemical mechanical polishing (CMP), the interposer channel uses meshed ground layer, so the AC resistance of the interposer is relatively high. Since the meshed layer has many slots, the return path is relatively longer than that in a solid ground layer in the printed circuit board (PCB). As shown in Figure 10b, the capacitance of the proposed channel structure is significantly reduced compared to that for the strip line. Since the proposed channel structure is arranged in the structure of the broadside, this structure can achieve the effect of minimizing the self-capacitance. As shown in Figure 10c, the self-inductance tends to be greater as there are more regions in which the magnetic field can be generated. Since the strip line is a closed structure due to the ground layer, the self-inductance is relatively small. Below 1 GHz, the proposed channel structure is smaller than that for the micro-strip line because it is a half-closed structure. However, as the frequency increases, the self-inductance becomes similar to that for the micro-strip line.

In terms of signal transmission, the impedance matching has a greater effect on signal transmission characteristics than the signal loss due to AC resistance at a high-frequency band. This is because the signal loss reduces the level of the signal, but the severe reflection due to impedance mismatching makes it impossible to transmit the signal normally. In the low-frequency band, the proposed structure can obtain good performance by reducing the resistance. The reason for this is that signal loss is more important than reflection in the low-frequency bands.

**Figure 11:**
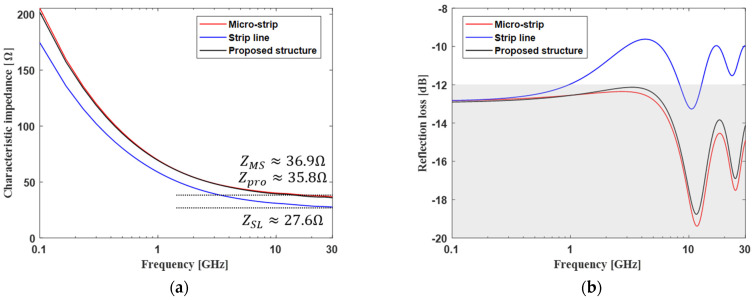
Impedance analysis of the proposed channel structure and conventional channel structure: (**a**) characteristic impedance and (**b**) reflection loss.

The results in Figure 11 confirm the impedance of the proposed channel structure and the conventional channel structure. We can estimate the characteristic impedance using (35) [21]. Based on the analysis of the RLC component, the proposed channel structure has a characteristic impedance similar to that for the micro-strip line, as shown in Figure 11a. Impedance matching was also checked through reflection loss, as shown in Figure 11b. If the reflection loss is less than 12 dB, it means that the reflection of the signal is sufficiently small due to the impedance mismatching for a driver impedance of 50 ohms. The strip line is not significantly impedance matched for some frequency bands under the same width and space channel conditions. On the other hand, it was confirmed that the proposed channel structure provides good impedance matching over a wide frequency band similar to that for a micro-strip line.

(35) $$Z_{0}=Z_{sys}\sqrt{\frac{{\left(1+S_{11}\right)}^{2}-S_{21}^{2}}{{\left(1-S_{11}\right)}^{2}-S_{21}^{2}}}$$

**Figure 12:**
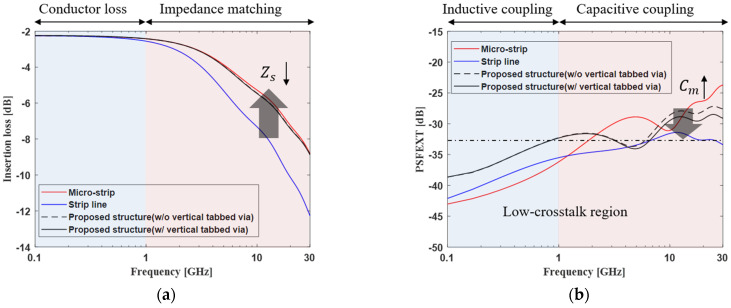
Frequency domain analysis of the proposed channel structure and conventional channel structure: (**a**) insertion loss and (**b**) PSFEXT.

The channel loss and far-end crosstalk of the proposed channel structure and conventional channel structure were compared, as shown in Figure 12a. First of all, it was confirmed that the proposed channel structure significantly improves the channel loss in broadband compared to the strip line, which is the worst case for a conventional structure. This effect comes from the improvement obtained by reducing the impedance mismatching.

Since the proposed channel structure has vertical symmetry, it shows almost the same performance regardless of the position of the interposer channel. Below 1 GHz, the conductor loss is dominant. The conductor loss is formed according to the AC resistance. The proposed channel structure has the smallest conductor loss because the effective conductor area is the largest. Above 1 GHz, the characteristic impedance is dominant according to the self-capacitance and self-inductance. Since the proposed channel structure and micro-strip line have similar values, it can be confirmed that, above 1 GHz, the channel loss is similar. On the other hand, the strip line has a larger capacitance than the others. It can be seen that the signal is greatly degraded above 1 GHz.

As shown in Figure 12b, in terms of far-end crosstalk, the PSFEXT of the strip line is the smallest of the channels in the overall frequency range. Since the strip line is located between the ground layers, it can be easily predicted that the far-end crosstalk noise is small. For the micro-strip line and the proposed channel structure, it can be confirmed that the far-end crosstalk is relatively large because the ground layer is located only on one side.

The proposed channel structure has structural advantages in terms of far-end crosstalk. This is because the proposed channel structure can generate more mutual capacitance than the micro-strip line due to the vertical tabbed via. In addition, by adding the vertical tabbed via, the influence of far-end crosstalk can be mitigated. The comprehensive evaluation including the channel loss and far-end crosstalk can be checked using the eye diagram in the next section.

**Figure 13:**
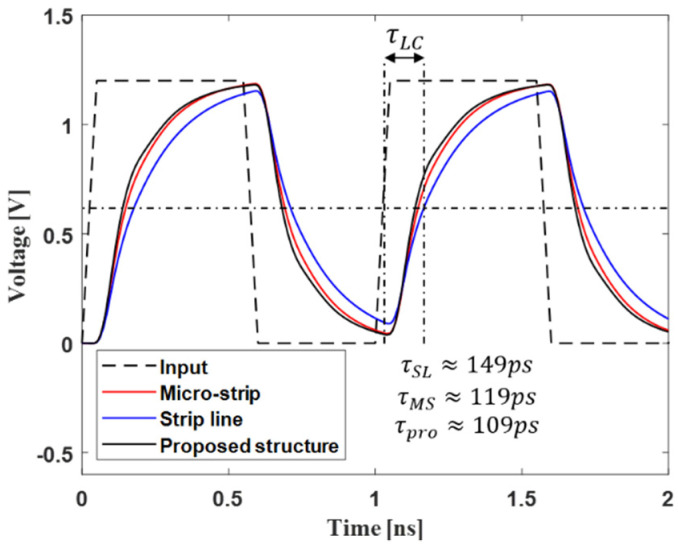
Timing analysis of proposed channel structure and conventional channel structure.

The proposed channel structure has an advantage in terms of timing. The effect of LC delay is also determined by the self-inductance and self-capacitance of the off-chip interconnection. Here, LC delay means a delay in the signal generated through the off-chip interconnection. As the LC delay of the channel increases, the ISI can also increase. The LC delay is defined as the difference in time at which the input waveform and output waveform have transitioned to about 50% of their maximum value, respectively. As shown in Figure 13, by minimizing the parasitic self-capacitance, the proposed channel structure can reduce the LC delay by about 20 ps compared to the strip-line, which is the worst case for the conventional structure.

### 4.2. Eye-Diagram and Dynamic Power Consumption Analysis of the Proposed Novel Interposer Channel Structure Compared with the Conventional Channel Structure

**Figure 14:**
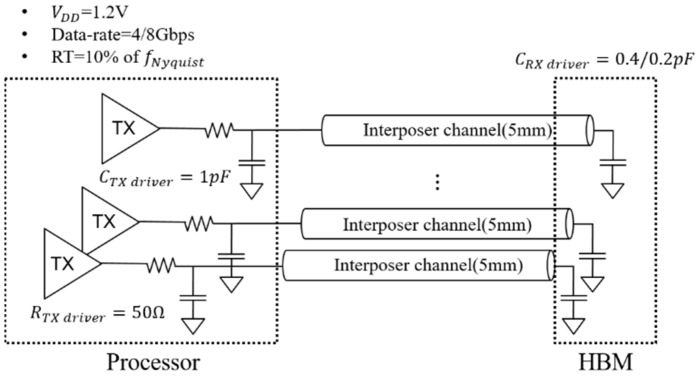
Eye-diagram simulation setup.

Using an eye diagram, it is possible to compare and analyze the signaling performance of the channel by comprehensively evaluating various signal integrity indicators. As shown in Figure 14, the eye-diagram setup refers to the JEDEC standard [22]. The data rate was evaluated for 4 and 8 Gbps, which are the performance levels of HBM Gen 3 and HBM Gen 4. The load of the TX driver includes the effects of electro-static discharge (ESD) and pad. The load of the RX driver does not have resistor termination due to the power consumption issue. Referring to the JEDEC standard, if the data rate is doubled, the load tends to be reduced by half. Therefore, the load value for 8 Gbps is assumed to be 0.2 pF. The selected rising time was as 10% of the Nyquist frequency.

**Figure 15:**
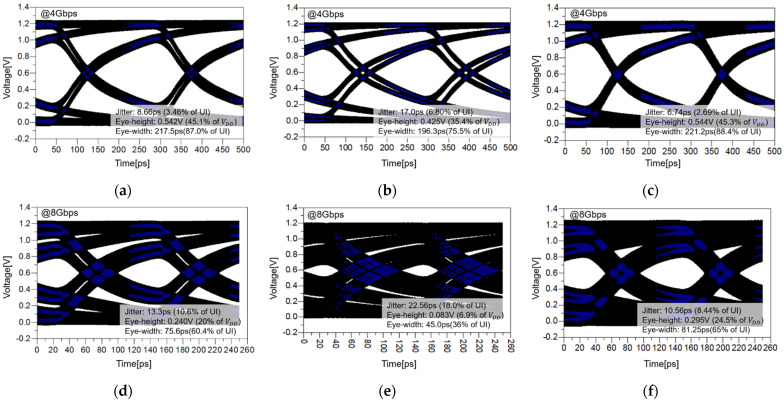
Eye-diagram of the conventional micro-strip line, strip line, and the proposed structure: (**a**) micro-strip line at 4 Gbps, (**b**) strip line at 4 Gbps, (**c**) proposed structure at 4 Gbps, (**d**) micro-strip line at 8 Gbps, (**e**) strip line at 8 Gbps, and (**f**) proposed structure at 8 Gbps.

The results of the eye diagram for 4 and 8 Gbps are shown in Figure 15. It can be seen that the results for impedance, insertion loss, far-end crosstalk, and eye diagram have the same tendency. In the eye diagram, the proposed channel structure shows similar or better performance to that for the micro-strip line. The strip line significantly degrades the signaling performance. In general, the system performance is determined based on the worst case. By using the proposed channel structure, the overall performance of the system can be surely improved. Since the proposed channel structure is a vertical symmetric structure, all channels have uniform performance. This result can be confirmed more clearly when the higher data rate 8 Gbps signal is transmitted. In the case of 8 Gbps, it can be seen that the strip line is almost closed in the eye-diagram. As the data-rate increases, it means that the strip line cannot transmit the signal.

At 4 Gbps, the proposed channel structure can improve eye width, eye height, and eye jitter by up to 12.9%, 9.9%, and 4.11%, respectively, compared to the worst-case, which is that for the strip line. At 8 Gbps, the channel loss of the worst-case can mean more degradation. The proposed channel structure can improve eye-width, eye-height, eye-jitter by up to 17.6%, 29%, and 9.56%, respectively, compared to the worst case.

**Figure 16:**
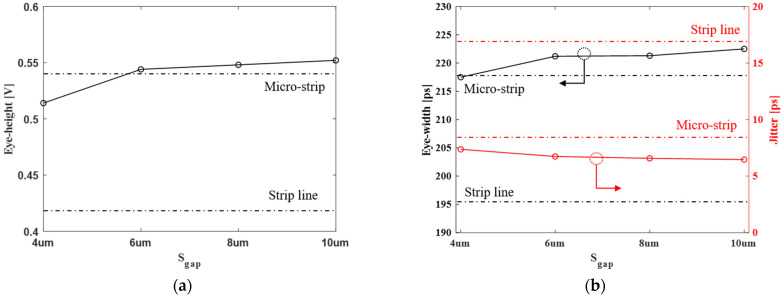
Summary of the eye-diagram data: (**a**) eye-height and (**b**) eye-width and jitter.

The eye margins are summarized, depending on the gap of the vertical channel, in Figure 16. We obtained additional eye-margin because the far-end crosstalk is improved as the gap in the vertical channel increases. However, as mentioned in the previous section, when the gap of the vertical channel is increased above a certain value, the amount of far-end crosstalk improvement can be limited.

Figure 16 indicates how much of a gap of the vertical channel needs to be selected to show a better eye margin than the conventional channel structure. The proposed channel structure shows better signaling performance than the micro-strip line when the vertical channel gap is more than 6 μm.

Additionally, the proposed channel structure has an advantage in terms of power consumption. As mentioned before, the power issue that occurs off-chip should be minimized. Power consumption can be calculated using (36) [23]. Based on the dynamic power consumption formula, the way to reduce power consumption in off-chip is to minimize parasitic capacitance [24]. The proposed channel structure forms a capacitance similar to that of micro-strip line regardless of the channel position. However, since the strip line forms a very large capacitance, the conventional channel structure consumes a lot of power. Compared with the average power consumption, it can be reduced up to about 28%. Table 2 summarizes the amount of power consumption depending on the channel structure and data rate.

(36) $$P_{dynamic}=\frac{1}{2}V_{DD}^{2}f_{Nyquist}C_{total}$$

Table 3 summarizes of the evaluation factors in terms of the signal integrity, and power consumption analyzed for the conventional structure and the proposed structure. The proposed structure obtains similar or better signaling performance than the micro-strip line in most evaluation factors, and the proposed structure is significantly improved compared to the strip line. Conventional structures should always be used together with the micro-strip and strip line. Thus, the strip line determines the worst performance of the conventional structure. However, since the proposed structure is vertically symmetrical, the signaling performance of all channels is more uniform than in the conventional structure. Therefore, the proposed structure is the advanced channel structure for the high-speed and low-power channels for the next-generation HBM.

## 5. Conclusions

In order to realize a next-generation HBM, a structural change to a broadside interposer channel structure is needed to alleviate the impedance mismatching problem. In this paper, to improve high-speed signaling and low-power consumption, a broadside interposer channel with vertical tabbed via is proposed and analyzed for the first time. Using the 3D EM simulation, we suggested and verified the analytical modeling of the self-capacitance and mutual capacitance for the proposed interposer channel depending on various design parameters. Based on the proposed analytical modeling, the insertion loss and far-end crosstalk of the proposed interposer channel were analyzed depending on the design parameters.

The proposed interposer channel structure has four major advantages. First, since the impedance mismatching can be minimized by arranging the broadside structure, the proposed interposer channel can dramatically improve channel loss. Second, by inserting the vertical tabbed via, the shortcoming of the far-end crosstalk issue in the broadside structure can be reduced. Third, the proposed interposer channel can minimize off-chip capacitance by about 28% compared to the conventional structure in terms of dynamic power consumption. Finally, the proposed interposer channel can prevent E-field and H-field from being emitted to the outside of the interposer by arranging a ground layer above and below. It is possible to prevent far-end crosstalk noise occurring between the interposer channel and other components such as TSV, via, etc. In addition, this effect can be achieved in terms of channel density because the number of the signal layer can be reduced in a situation that requires an additional signal layer. Although there is a difference in performance depending on $s_{gap}$, eye-width, height, and jitter were improved by 17.6%, 29%, and 9.56%, respectively, at 8 Gbps compared to a strip line based on 6 µm.

The proposed interposer channel is expected to be used as a high-speed interconnection channel structure to stably transmit a higher data rate in the future. In addition, it can solve the issue of power consumption.

## Figures and Tables

**Table 1:** Physical dimensions and material properties of the silicon interposer.

Symbol	Parameter	Value	Symbol	Parameter	Value
$w_{SIG}$	Width of channel	3 μm	$w_{GND}$	Width of a meshed ground layer	3 μm
$s_{SIG}$	Space of channel	3 μm	$s_{GND}$	Space of a meshed ground layer	3 μm
$h_{sub}$	Height of Si substrate	100 μm	$l_{channel}$	Length of channel	5 mm
$t_{RDL}$	Height of RDL layer	1 μm	$\varepsilon_{Si3N4}$	$\mathrm{Relative}\;\mathrm{permittivity}\;\mathrm{of}\;Si_{3}N_{4}$	6.5
$h_{SiO2}$	$\mathrm{Height}\;\mathrm{of}\;SiO_{2}$ layer	1 μm	$\varepsilon_{SiO2}$	$\mathrm{Relative}\;\mathrm{permittivity}\;\mathrm{of}\;SiO_{2}$	4.1
$h_{pass}$	Height of passivation layer	1 μm	$tan\;\delta_{Si3N4}$	$\mathrm{Loss}\;\tan\mathrm{gent}\;\mathrm{of}\;Si_{3}N_{4}$	0.001
$s_{gap}$	Gap of the vertical channel	Design parameter (4 μm/6 μm/8 μm/10 μm)	$tan\;\delta_{SiO2}$	$\mathrm{Loss}\;\tan\mathrm{gent}\;\mathrm{of}\;SiO_{2}$	0.001
$h_{via}$	Height of vertical tabbed via	Design parameter (0.5 μm/1 μm/2 μm)	$\varepsilon_{Si}$	Relative permittivity of Si	11.9
$d_{via}$	Diameter of vertical tabbed via	1.5 μm	$\sigma_{Si}$	Conductivity of Si substrate	10 σ/m
$p_{via}$	Pitch of vertical tabbed via	Design parameter (3 μm/9 μm/18 μm)	$\sigma_{Cu}$	Conductivity of copper	$5.8\times 10^{7}\;\sigma /m$

**Table 2:** Dynamic power consumption of proposed structure and conventional channel structure.

Parameter	Conventional Micro-Strip	Conventional Strip Line	Proposed Structure
$C_{\mathrm{channel}}\;$[pF/mm]	0.158	0.294	0.163
$P_{\mathrm{channel}}\;$[mW/mm] at 4 Gbps	0.227	0.423	0.234
$P_{\mathrm{channel}}\;$[mW/mm] at 8 Gbps	0.455	0.846	0.469
$P_{\mathrm{average}\;\mathrm{channel}}\;$[mW/mm] at 4 Gbps	0.325		0.234 (−28%)

**Table 3:** Summary of system performance of the proposed and conventional structure.

Parameter	Conventional Micro-Strip	Conventional Strip Line	Proposed Structure
Reflection loss [dB] at 4 GHz	−12.5	−9.62	−12.19
Inseriton loss [dB] at 4 GHz	−3.50	−4.68	−3.55
FEXT [dB] at 4 GHz	−29.09	−33.83	−33.72
Characteristic impedance [Ω] at 4 GHz	45.6	36.4	45.2
LC delay [ps]	119	149	109
Eye-width [ps]	75.6	45.0	84.3
Eye-height [V]	0.240	0.083	0.295
Power consumption [mW/mm] at 8 Gbps	0.455	0.846	0.469

## Data Availability

The data presented in this study are available on request from the corresponding author.
